# An Impressive Advancement in Neuro-Oncology in Pakistan

**DOI:** 10.12669/pjms.41.13(PINS-NNOS).14601

**Published:** 2025-12

**Authors:** Syed Ather Enam*

**Affiliations:** Syed Ather Enam, MD, PhD, FRCSI, FRCSC, FRCSG, FACS Professor of Neurosurgery, Aga Khan University Hospital, President, Pakistan Association of Neuro-Oncology, Sakar Khanum & Hussain Ebrahim Family Chair Director, Centre for Regenerative Medicine and Stem Cell Research Director, Centre of Oncological Research in Surgery Scientific Director, Juma Research Laboratories

The field of Neuro-Oncology in Pakistan is gaining more attention as we advance in neurosurgery. Despite significant challenges, notable progress is being made. This is the first-ever Neuro-Oncology Supplement stemming from efforts by a public sector institution. It reflects an impressive advancement in Neuro-Oncology in Pakistan, as the problems faced by patients with brain tumors in Pakistan are most severe when they present at a public sector institution. This supplement, produced by the Pakistan Journal of Medical Sciences (PINS–NNOS 2025), is a remarkable achievement in this context. It highlights the tireless efforts of the Punjab Institute of Neurosciences (PINS) and its role in becoming one of the leaders in neuro-oncology in the country. This supplement will help us identify key aspects, both the progress made and the gaps that remain, to improve care, research, and education in this critical field. The editorial rigor seen in this supplement is of a very high standard. The multi-stage, double-blind peer review process reflects the growing academic maturity in Pakistan.

The breadth of submissions in this supplement makes it stand out and reflects the national diversity of contributors as well as the interdisciplinary nature of neuro-oncology emerging in Pakistan. Contributions encompass various neuro-oncology subspecialties, including neurosurgery, oncology, histopathology, and rehabilitation medicine. We will highlight a few studies, such as “Improving Final Diagnosis in Paediatric Posterior Fossa Tumors”,^1^ which exemplifies the importance of combining diagnostic methods in Pakistan—especially given the limited availability of multidisciplinary tumor boards in many regions across the country.

The supplement also highlights significant challenges, including resource limitations and limited access to specialized care. “Determinants of Poor Prognosis after Surgery for Glioblastoma”^2^ offers valuable insights into factors that contribute to high morbidity and mortality in glioblastoma surgery, emphasizing the importance of preoperative care and the extent of resection for improving survival outcomes in low- and middle-income countries (LMICs). Studies emphasize that even with aggressive treatment, recurrence remains a significant challenge, and further research is necessary to understand the factors influencing patient outcomes.

A significant issue, which I am glad was accepted for publication in this supplement by independent peer review process, is the lack of training and exposure to surgical neuro-oncology among many neurosurgical residents nationwide. In the “Clinical Landscape and Perception of Surgical Neuro-Oncology among Neurosurgical Resident Trainees of Pakistan,”^3^ while most residents have a solid theoretical understanding of brain and spinal tumors, they often lack sufficient hands-on experience and access to mentoring.

Establishing dedicated neuro-oncology fellowships nationwide would significantly boost the clinical and academic capabilities of Pakistani neurosurgeons. Besides training, interdisciplinary collaboration remains a challenge, with limited opportunities for meaningful engagement among different specialties.

The supplement’s focus on including manuscripts from various subspecialties highlights that neuro-oncology care extends beyond surgery. Radiology, palliative care, and rehabilitation medicine are equally essential in providing comprehensive patient care. The study on “Impact of Neurorehabilitation on Functional Recovery in Patients with Intradural Spinal Cord Tumors” emphasizes the importance of structured rehabilitation programs in enhancing patient outcomes after spinal tumor surgery, especially in resource-limited settings.^4^

This holistic approach is especially crucial in LMICs, where multidisciplinary resources might not always be easily accessible. Going forward, it will be vital for Pakistan’s neuro-oncology community to strengthen these partnerships and ensure that patients receive comprehensive, patient-centered care.

Looking ahead, we need to focus on two key areas: building a strong academic culture and improving patient outcomes through evidence-based research. The success of initiatives like this can motivate more residents, young faculty, and institutions to get involved in scientific publishing, research mentorship, and data collection efforts. By enhancing our academic infrastructure, we can ensure that neuro-oncology in Pakistan is not only recognized for its accomplishments but also prepared to meet future challenges.

This supplement stands as a testament to the vision and perseverance of Prof. Asif Bashir, whose leadership at the Punjab Institute of Neurosciences has created an enabling environment for academic growth within the public sector, and to the remarkable dedication of Dr. Haseeb Mehmood Qadri and his team, whose tireless coordination, editorial stewardship, and meticulous attention to detail guided this project from conception to completion. Dr. Haseeb stands out as an exceptionally dedicated and inspirational young neurosurgery resident whose unwavering devotion to advancing public-sector neurosurgery reflect a maturity far beyond his years of training. Together, their mentorship and commitment exemplify the spirit of collaboration and the promise of a new generation of academic leadership in neuro-oncology in Pakistan.

The partnership between PASNO (Pakistan Society of Neuro-Oncology) and PINS marks an important step toward strengthening the foundation of neuro-oncology in Pakistan. As PASNO evolves into a unifying national platform for advancing research, training, and policy in neuro-oncology, its collaboration with a leading public-sector institution like PINS ensures that these goals are translated into tangible clinical and academic outcomes. PINS, with its patient volume, multidisciplinary structure, and academic vision, is uniquely positioned to serve as the institutional anchor for implementing PASNO’s mission of improving access, quality, and collaboration in neuro-oncology across the country.

The PINS–NNO 2025 Supplement is a team effort to expand neuro-oncology care and research in Pakistan. It also acts as a call to action for greater collaboration, mentorship, and resource support. Together, we can continue shaping the future of neuro-oncology in Pakistan and beyond.

We thank all contributors and reviewers and look forward to the ongoing success of this national effort.
